# Optimization and Formulation of Fucoxanthin-Loaded Microsphere (F-LM) Using Response Surface Methodology (RSM) and Analysis of Its Fucoxanthin Release Profile

**DOI:** 10.3390/molecules24050947

**Published:** 2019-03-07

**Authors:** Irwandi Jaswir, Dedi Noviendri, Muhammad Taher, Farahidah Mohamed, Fitri Octavianti, Widya Lestari, Ali Ghufron Mukti, Sapta Nirwandar, Bubaker B. Hamad Almansori

**Affiliations:** 1 International Institute for Halal Research and Training (INHART), International Islamic University Malaysia, Jalan Gombak, Kuala Lumpur 53100, Malaysia; dokterwidya@gmail.com (W.L.); science.abu@gmail.com (B.B.H.A.); 2 Bioprocess and Molecular Engineering Research Unit (BPMERU), International Islamic University Malaysia (IIUM) Gombak, Kuala Lumpur 53100, Malaysia; ir98@hotmail.com; 3 Department of Pharmaceutical Technology, Universitas Ahmad Dahlan, Yogyakarta 55164, Indonesia; 4 Department of Pharmaceutical Technology, Faculty of Pharmacy, International Islamic University Malaysia Kuantan, Kuantan 25200, Malaysia; mtaher@iium.edu.my (M.T.); farahidah@iium.edu.my (F.M.); 5 Department of Orthodontics, Faculty of Dentistry, Universiti Sains Islam Malaysia, Tower B, Persiaran MPAJ, Jalan Pandan Utama, Kuala Lumpur 55100, Malaysia; yanti75@yahoo.com; 6 Ministry of Research, Technology and Higher Education of Indonesia, Senayan, Jakarta Pusat 10340, Indonesia; ghufronmukti@yahoo.com; 7 Chairman, Indonesia Halal Lifestyle Foundation, Jakarta 10230, Indonesia; bob.naedi@gmail.com

**Keywords:** fucoxanthin, release profile, response surface methodology, microsphere, microencapsulation

## Abstract

Fucoxanthin has interesting anticancer activity, but is insoluble in water, hindering its use as a drug. Microencapsulation is used as a technique for improving drug delivery. This study aimed to formulate fucoxanthin-loaded microspheres (F-LM) for anticancer treatment of H1299 cancer cell lines and optimize particle size (PS) and encapsulation efficiency (EE). Using response surface methodology (RSM), a face centered central composite design (FCCCD) was designed with three factors: Polyvinylalcohol (PVA), poly(d,l-lactic-co-glycolic acid) (PLGA), and fucoxanthin concentration. F-LM was produced using a modified double-emulsion solvent evaporation method. The F-LM were characterized for release profile, release kinetics, and degradation pattern. Optimal F-LM PS and EE of 9.18 µm and 33.09%, respectively, with good surface morphology, were achieved from a 0.5% (*w*/*v*) PVA, 6.0% (*w*/*v*) PLGA, 200 µg/mL fucoxanthin formulation at a homogenization speed of 20,500 rpm. PVA concentration was the most significant factor (*p* < 0.05) affecting PS. Meanwhile, EE was significantly affected by interaction between the three factors: PVA, PLGA, and fucoxanthin. In vitro release curve showed fucoxanthin had a high burst release (38.3%) at the first hour, followed by a sustained release stage reaching (79.1%) within 2 months. Release kinetics followed a diffusion pattern predominantly controlled by the Higuchi model. Biodegradability studies based on surface morphology changes on the surface of the F-LM, show that morphology changed within the first hour, and F-LM completely degraded within 2 months. RSM under FCCCD design improved the difference between the lowest and highest responses, with good correlation between observed and predicted values for PS and EE of F-LM.

## 1. Introduction

Fucoxanthin, a major xanthophylls in brown seaweed, has a unique structure, including a usual allenic bond and 5,6-monoepoxide in its molecule. Fucoxanthin has been reported to induce apoptosis in prostate cancer PC-3, DU145 and LNCap cells, leukemia HL-60 cells, and caused cell cycle arrest during the G_0_/G_1_ phase in neuroblastoma GOTO cells [1] Furthermore, in colon cancer cell lines, fucoxanthin has been shown to induces apoptosis in Caco-2, HT-29 and DLD-1 cells [1,2,3,4].

Health care systems is Japan, Korea, and India use fucoxanthin for many applications, such as anticancer, antiobesity, antidiabetic, to induce cell cycle arrest at G_0_/G_1_ phase in human colon carcinoma cells, antioxidant, and anti-inflammatory applications. This compound can be isolated from varying types of brown seaweed, such as *Undaria pinnatifida*, *Hijkia fusiformis*, *Sargassum fulvellum*, and *Laminaria japonica*, from Japan, *Padina tetrastomatica* from India, and *Sargassum siliquastrum* from Korea, as well as *T. turbinata* and *S. plagyophyllum* from the Malaysian Peninsular [2,5,6,7].

Fucoxanthin has the potential to become an anticancer drug candidate due to its anticancer activities. Unfortunately, this carotenoid is insoluble in water, which poses a problem for its use as a drug candidate. Microencapsulation (ME) is one of the most interesting drug delivery systems [1], which can delay and modify drug release from pharmaceutical dosage forms [2]. Many studies have reported the advantages of formulation in the delivery of insoluble drugs using ME [3,4,5,6].

RSM is the collection of statistical techniques useful for developing, improving, and optimizing processes [8]. RSM defines the effect of the independent variables, alone or in combination, on the process [9]. The main advantage of RSM is to reduce the number of experimental trials needed to evaluate multiple variables [10] and its interactions; it is less laborious and time-consuming than other approaches [11,12].

The objective of this study was to optimize and formulate fucoxanthin-loaded microspheres (F-LM) for anticancer treatment of the human lung cancer (H1299) cell line using response surface methodology (RSM). Particle size (PS) and encapsulation efficiency (EE) of the F-LM fabricated via microencapsulation technique and fucoxanthin release profile, release kinetics, and its degradation.

## 2. Results and Discussion

### 2.1. Optimization of Microencapsulation Component by RSM

A face centered central composite design (FCCCD) under RSM was used to investigate the optimal conditions of the three significant factors (PVA, PLGA, and fucoxanthin concentration) towards PS and EE for the anticancer treatment on the human lung cancer (H1299) cell line. For each run, the experimental (observed) results, along with the predicted PS and EE obtained from the regression equations for the 15 combinations, are shown in Table 1.

The results demonstrated that optimal PS and EE, by double emulsion extraction/evaporation method (9.18 µm and 33.09%), were observed in the runs representing the center point (run 12). This size is desirable for pulmonary drug delivery, namely between 1 to 10 µm. Furthermore, the highest amounts of PS and EE (10.95 µm and 34.87%, respectively), by this method, were observed in the run representing the center point (run 11) and the lowest amounts were observed in run 15 (2.01 µm and 10.25%), where factors such as PLGA and fucoxanthin were at the lowest concentrations, whereas the PVA concentration was at the highest concentration. In this study, Table 2 shows the design matrix of FCCCD, which further improved the PS and EE, such that the difference between the lowest and the highest response was (2.01 to 10.95 µm; 10.25% to 34.87%).

A second order regression Equation showed the dependence of PS and EE of F-LM produced by double emulsion extraction/evaporation method on the microencapsulation components. The parameters of the equation were obtained by multiple regression analysis of the experimental data [13]. An empirical relationship between the screened and response variables were expressed in terms of the second-order polynomial equation:

*Y*_1_ (PS, µm) = + 9.30 − 1.16*A* + 0.83*B* + 0.45*C* −3.76*A*^2^ − 0.42*B*^2^ − 0.95*C*^2^ + 0.20*AB* − 0.085*AC* + 0.20*BC* (1)

where the PS is the response (*Y*_1_) and *A*, *B*, and *C* are the concentrations of PVA, PLGA, and fucoxanthin, respectively.

*Y*_2_ (EE, %) = +32.34 − 2.69*A* + 3.51*B* + 5.06*C* − 0.72*A*^2^ − 2.43*B*^2^ − 4.93*C*^2^ + 0.35*AB* + 0.77*AC* − 1.06*BC* (2)

where the EE is the response (*Y*_2_) and *A*, *B*, and *C* are the concentration of PVA, PLGA, and fucoxanthin, respectively.

The adequacy of the model for PS and EE were checked using ANOVA. which was tested using Fisher’s exact test and the results are shown in Table 2 and Table 3. For PS (Table 2) the model F value of 8.99 and a *p*-value of <0.0131 imply that the model is significant, suggesting that there is only 1.31% chance that the model F value could occur due to noise. Model terms with a probability >F (less than 0.05) are considered significant, while those greater than 0.10 are insignificant [13]. Furthermore, for EE (Table 3), the model F value of 23.17 and a *p*-value of <0.0015 imply that the model is significant, suggesting that there is only 0.15% chance that the model F value could occur due to noise. The model terms with a probability >F (less than 0.05) are considered significant [14].

**Figure 1:**
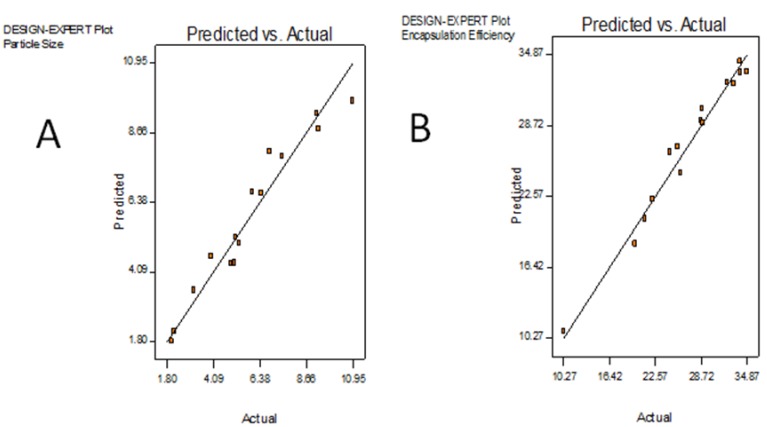
The design experts plot between predicted and actual values for PS (particle size) (**A**) and EE (encapsulation efficiency) (**B**).

An R^2^ value closer to 1 denotes better correlation between the experiment (observed) and predicted values. For PS (Table 3 and Figure 1A), the higher values of R^2^ (0.9418) and adjusted R^2^ (0.8371) also indicated the efficacy of the model and 94.18% or 83.71% variations could be accounted for by the model equation. Thus, for a good statistical model, the R^2^ value should be in the range 0 to 1.0, and the closer the value is to 1.0, the better fit the model is deemed to be [14]. Moreover, adequate precision measures signal to noise ratio, and a value >4 is considered a prerequisite for desirable models [13]. The adequate precision value of 9.249 for PS indicates that the model can be used to navigate the design space. Furthermore, for EE (Table 4 and Figure 1B), the higher values of R^2^ (0.9766) and adjusted R^2^ (0.9344) also indicated the efficacy of the model, and 97.66% or 93.44% variations could be accounted for by the model equation. The adequate precision value of 16.752 for EE indicates that the model can be used to navigate the design space.

The correlation coefficient values of regression equation are listed in Table 2 and Table 3. The *p*-value is used as a tool to check the significance of each coefficient [14], which also indicates the interaction strength between each independent variable. The smaller the *p*-value, the bigger significance of the corresponding coefficient. For PS (Table 2), the responses revealed that only one interaction term (*A*-PVA), and one the quadratic coefficient (*A*^2^) were significant (*p* < 0.05), and had remarkable effects on the overall PS.

Lakshmana et al. [15] and Dhakar et al. [16] reported that the PS of microsphere is seen to be dependent on the concentration of PVA in the continuous phase. From this study, the results revealed that, with an increase in the concentration of PVA, more PVA molecules may overlay the surface of the droplets. Increasing of the concentration PVA provides conditions to obtain smaller emulsion droplets [17]. Furthermore, increasing PVA concentration has been shown to provide an increased protection of the droplets against coalescence resulting in the production of small PS [17,18,19].

For EE (Table 2), the response for one interaction term (*A*-PVA), (*B*-PLGA), and (*C*-fucoxanthin) and the quadratic coefficient, were significant (*p* < 0.05) and had remarkable effects on the overall EE. Ruan and Feng [20] reported that the EE is defined as the ratio of amount of encapsulated drug to that of the drug used for microsphere preparation. Dhakar et al. [16] reported that the loading efficiency of drug release from the microsphere depended on the concentration of polymer and type of polymer used. EE has been shown to increase alongside an increasing concentration of polymer [21]. The high polymer concentration results in large microspheres, which causes more loss of drug from the surface during washing of the microspheres compared to smaller microspheres [16]. Thus, microsphere size also affected EE [16].

**Figure 2:**
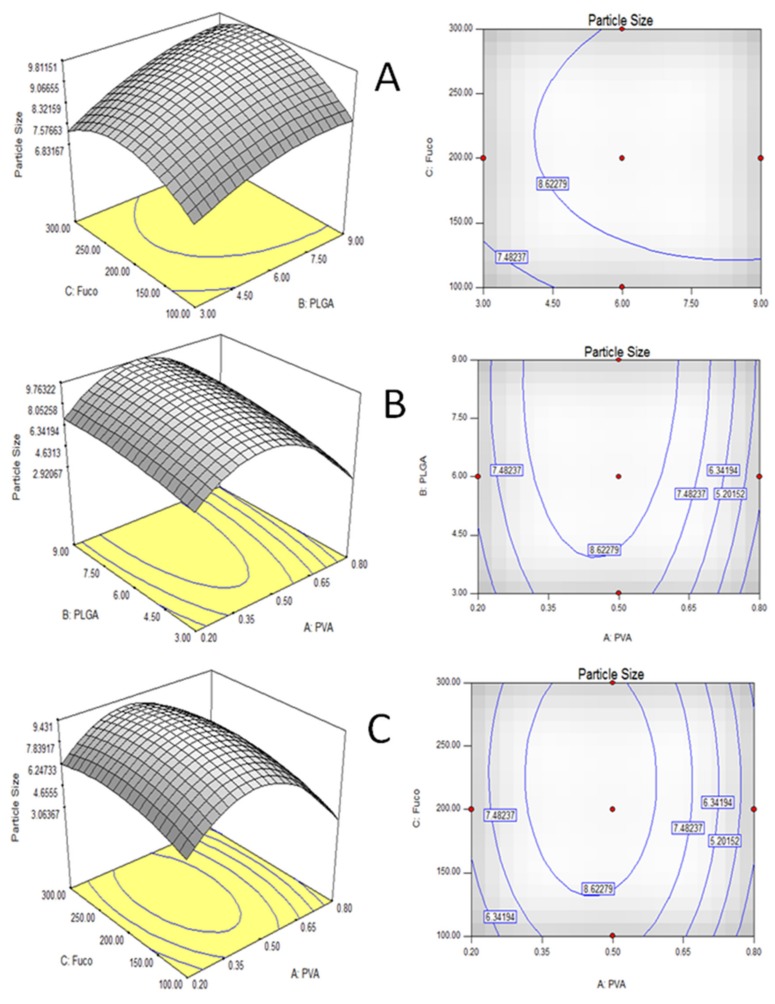
3D response surface curves and 2D contour of the combined effects of PVA (polyvinylalcohol), poly lactic-co-glycolic acid (PLGA), and fucoxanthin concentration on PS (particle size) by double emulsion extraction/evaporation method (**A**) PLGA and fucoxanthin at fixed level of PVA; (**B**) PVA and PLGA at fixed level of fucoxanthin; (**C**) PVA and fucoxanthin at fixed level of PLGA.

The 3D response surface plot is the graphical representation of the regression equation used to investigate the interactions among variables and to determine the optimum concentration of each factor [12] for optimum PS and EE by *w*/*o*/*w* double emulsion extraction/evaporation method. The 3D response surface and contour plots of the combined effects of PVA, PLGA, and fucoxanthin concentration for PS and EE by double emulsion extraction/evaporation method are shown in Figure 2. The 3D plots are based on the function of concentrations of two variables, with the other variable being at its optimum level [9]. Significant interaction between the corresponding variables is indicated by an elliptical or saddle nature of the contour plots [12,21,22,23]. Figure 2A represents the interaction between PLGA (coating) and fucoxanthin (core) concentration. Lower and higher levels of both the PLGA and fucoxanthin did not result in higher PS. Figure 2B shows the 3D plot corresponding to PVA and PLGA concentration. In the case of PVA and fucoxanthin (Figure 2C), the response plot was elliptical, showing interaction between them with optimum PS by double emulsion extraction/evaporation method.

Furthermore, the elliptical response plot in Figure 2A shows interactions between PLGA and fucoxanthin with optimum EE by double emulsion extraction/evaporation method, whereas Figure 2B,C show the 3D plots corresponding to PVA and PLGA, and PVA and fucoxanthin concentration, respectively. Lower and higher level PVA and PLGA, and PVA and fucoxanthin did not result in higher EE.

### 2.2. Particle Size (PS), Size Distribution, and External Morphology of F-LM

Based on an earlier study, a homogenization speed of 20,500 rpm was successfully used to fabricate the F-LM particle size <10 µm (desirable size). Thus, a high speed homogenization (20,500 rpm) was employed for further study.

**Figure 3:**
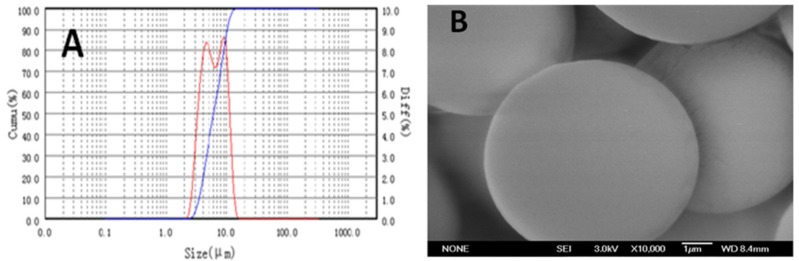
A representative of PS distribution of fucoxanthin-loaded microspheres (F-LM) by using Laser Particle Size (LPS) analyzer (BT-9000H, Bettersize Instrument Ltd., China) (**A**), a representative of external morphology of F-LM by using Field Emission Scanning Electron Microscope (FE-SEM) (JEOL, JSM 6700F Model, Japan) with magnification 10,000× (**B**).

From this study, Figure 3A shows the representative F-LM size distribution by laser particle sixe (LPS) analyzer. The F-LM size distribution was a narrow curve corresponding to uniform sizes, approximately 9 µm. This PS (9.18 µm) was achieved from the formulation of microsphere with 0.5% (*w*/*v*) PVA, 6.0% (*w*/*v*) PLGA, and 200 µg/mL fucoxanthin composition. From this study, fabrication of F-LM by using 0.5% (*w*/*v*) PVA as surfactant, 6.0% (*w*/*v*) PLGA as a coating, and 200 µg/mL fucoxanthin as a core, produced discrete spheres with smooth surfaces and no pore size (Figure 3B). Hong et al. [24] reported that the size distribution, PS, and pore size within the microspheres are influenced by fabrication conditions such as the concentration of polymer, stirring rate, good solvent/non solvent ratio, and the concentration of the dispersant.

### 2.3. In Vitro Fucoxanthin Release Profile of F-LM

To investigate the effect of outer aqueous phase composition of F-LM on the in vitro fucoxanthin release behavior of F-LM, a release test of F-LM in 0.1 M PBS (pH 7.4) at 37 °C in static conditions was performed. In vitro release was performed in Phosphate Buffered Saline (PBS) at pH 7.4 (bronchial pH) and not at pH 5.2 (alveolar pH) [23] because the acidic pH can accelerate degradation of PLGA, resulting in a reduced pH of the microenvironment [24].

**Figure 4:**
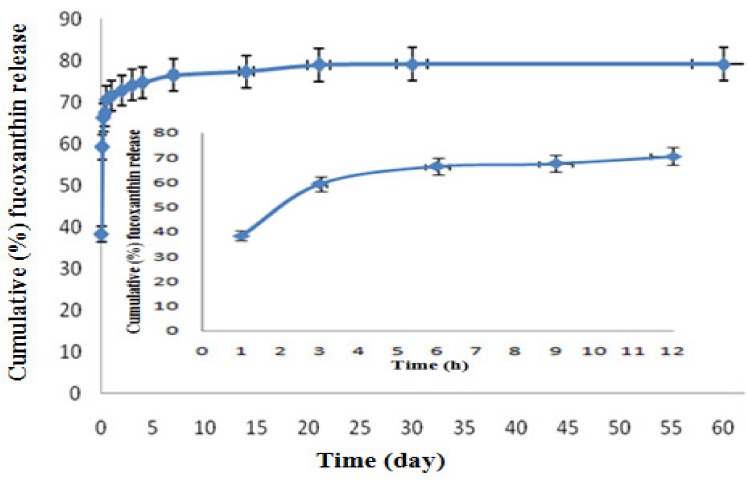
The curve of in vitro fucoxanthin release profile of F-LM as a function of time. In vitro fucoxanthin release was assessed by intermittently sampling the vial (1 mL) at predetermined time intervals (1, 3, 6, 9, and 12 h, then 1, 2, 3, and 4 days, then 1, 2, and 3 weeks, then 1 and 2 months), and was replaced with 1 mL of fresh 0.1 M PBS (pH 7.4) at 37 °C.

**Figure 5:**
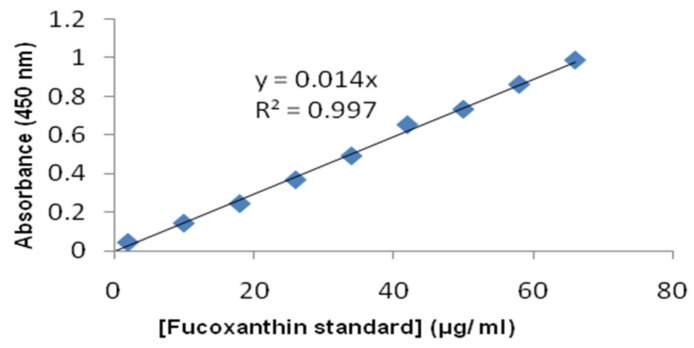
Standard curve of commercial fucoxanthin (purity >95%).

Figure 4 shows the in vitro fucoxanthin release profile of F-LM, and Figure 5 show a standard curve of fucoxanthin. The in vitro fucoxanthin release of F-LM was calculated based on this standard curve. Figure 4 represents the interaction between time intervals and cumulative (%) fucoxanthin release. The curve of in vitro fucoxanthin release showed two profiles: A rapid release (burst release) followed by a sustained release stage. Initially, a large burst release (38.3%) occurred at the first hour. This effect may be associated with the presence of fucoxanthin crystals on or nearby the surface of microspheres. A burst release was observed in this study because the polymer precursor did not set immediately, causing unsuccessful encapsulation of some fucoxanthin, thus allowing free fucoxanthin to release in a burst. The burst release is usually caused by fast desorption of the drug at the surface [17]. Subsequently, high burst release is attributed to the microsphere porous structure, which is commonly produced in the double *w*/*o*/*w* emulsion method [25,26,27]. In this case, the method used in this study was the *w*/*o*/*w* double emulsion extraction/evaporation method. However, the burst release was significantly reduced by the immediate lyophilization of the harvested microspheres following sonication-prepared emulsion [25].

Furthermore, in this study, the burst release step was followed by a gradual release of fucoxanthin, reaching (79.1%) within 2 months. The F-LM had a high burst release (38.3%) due to PLGA (50/50) used, and the small size of the microspheres. Tsai [26] reported that the microspheres with the lowest molecular weight (MW) PLGA (50/50) showed the highest initial burst release compared to higher MW of PLGAs (65/35; 75/25; or 85/15) microspheres [28] due to higher hydrophobic -CH_3_ of lactic acid (LA) in the composition.

The size ~9 µm of microspheres in this study affected the initial burst release of fucoxanthin. Makadia and Siegel [29] reported that the size of PLGA microsphere also affected the initial burst release. The drug release of smaller microspheres is faster than larger microspheres due to a higher surface area-to-volume ratio [13,30]. Increased surface area enhances polymer and fucoxanthin exposure to aqueous media, resulting in a larger initial burst and enhanced polymer degradation. Moreover, the smaller microspheres have a shorter diffusion path length [13,30], thereby increasing their penetration by the aqueous media [13,27].

Zhang and Zhu [31] have reported that, generally, microspheres prepared under various conditions displayed similar release profiles, such as burst release, followed by a sustained release stage. The initial burst release from microspheres might be due to the rapid release of the drug deposited on the microsphere surface [32]. This phenomenon occurs through the dissolution of the drug, present at or near microsphere surfaces [13,27]. The initial burst of drug release is related to the type of drug, the hydrophobicity of the polymer, and the concentration of drug [29].

Lewis [33] and Mao et al. [34] reported that the drug release pattern from PLGA microspheres was biphasic, as a combination of the simple diffusion of the drug out of the polymer matrix and erosion or degradation of the polymer matrix [35,36], which occurs via hydrolysis of the polymer back bone [37]. Initially, the drug is released via diffusion through the polymer matrix, as well as through the porous voids of the polymer structure [38]; but biodegradation of PLGA continuously changes the drug release pattern [32]. The second process involves bulk erosion: The polymer matrix uptakes water and polymer chains are degraded small enough to be soluble, and the drug is released during the dissolution of the PLGA matrix [39].

It is well known that drug substances near the surface will diffuse out the microsphere first, causing release [32]. The pattern of the result from this study was similar to the results obtained by Emami [40], where insulin showed higher burst release (28%) and lower encapsulation efficiency (44%) in PLGA microsphere prepared by *w*/*o*/*w* method.

### 2.4. Release Kinetics of F-LM

**Figure 6:**
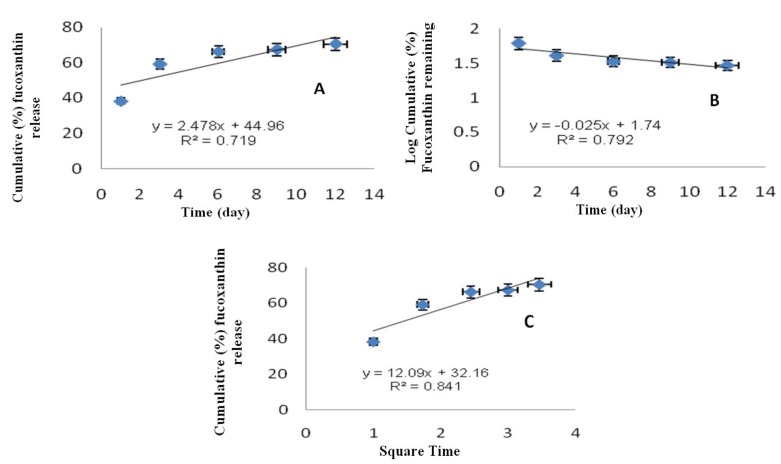
Zero-order kinetic data (**A**), first-order kinetic data (**B**), and Higuchi equation data (**C**) of F-LM.

In order to determine the release model which best describes the pattern of fucoxanthin release from F-LM, the in vitro fucoxanthin release data were substituted in zero order, first order, and Higuchi model. Figure 6 shows the zero order, first order, and Higuchi model of F-LM. Zero-order kinetics describe a system where fucoxanthin release rate is independent of concentration and its cumulative amount percentage of fucoxanthin release versus time (Figure 6A). The first-order kinetics describe the release rate of fucoxanthin as dependent of concentration and its cumulative percentage of fucoxanthin remains in the log scale versus time (Figure 6B). Higuchi model describes the release of fucoxanthin from an insoluble matrix as a square root of time dependent process (Figure 6C).

To confirm the pattern of fucoxanthin release, in vitro fucoxanthin release was subjected to release kinetic studies based on its respective R^2^ values, as given in Table 4. In this study, the in vitro release kinetic of F-LM was best explained by the Higuchi equation, as the plots showed linearity (R^2^ = 0.841), first order (R^2^ = 0.792), followed by zero order equation (R^2^ = 0.719). The best fit, with the highest correlation in Higuchi equation, indicated that the release follows a diffusion-controlled pattern [41]. Thus, from this result, it is concluded that the release of fucoxanthin from PLGA (50/50) matrix was predominantly controlled by Higuchi model.

### 2.5. Degradation Study of F-LM

**Figure 7:**
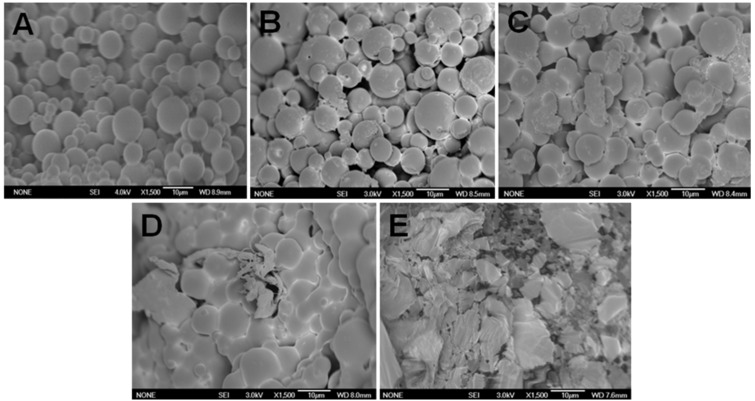
External morphologies change of F-LM dependent of time interval such as; control (**A**), 1 day (**B**), 1 week (**C**), 1 month (**D**), and 2 months (**E**) by using FE-SEM (JEOL, JSM 6700F Model) with magnification 1500×.

Biodegradability analysis of F-LM was based on changes in surface morphology of the microsphere. The changes in surface morphology of F-LM over time following incubation in 0.1 M PBS (pH 7.4) at 37 °C under static conditions is shown in Figure 7. An electron micrograph of F-LM before incubation (control) showed a spherical, discrete microsphere with a smooth surface (Figure 7A).

As illustrated in Figure 7B, minor changes in F-LM morphology were observed during the first hour. F-LM showed progressive pores emerging on the surface that describe the burst release phase. Following this, major changes in F-LM morphology were observed within 1 week and the F-LM had deformed, aggregated, and fused (Figure 7C), and by 1 month (Figure 7D), the F-LM morphologies had turned into an unstructured mass. Finally, the F-LM were totally collapsed and disintegrated into irregular particles, with no intact spheres observed (Figure 7E). The F-LM fabricated from PLGA (50/50) as coating, needed 2 months for degradation. Lewis [33] reported that controlled release of a desired drug is over a period of 1 to 3 months. Vidyavathy and Ramana [42] and Middleton and Tipton [43] reported that PLGA (50/50) polymer degraded in approximately 1 to 2 months, and PLGA 75/25 and PLGA 85/15 degraded in 4 to 5 months and 5 to 6 months, respectively.

PLGA polymer can be degraded into oligomeric and finally monomeric acids [42,43,44], such as lactic acid and glycolic acid that are non-toxic to the human body [45], and can be completely biodegraded into CO_2_ and H_2_O [46]. Furthermore, in the human body, polyglycolides are broken down into glycine which can be excreted in the urine or converted into CO_2_ and H_2_O via the citric acid cycle [47,48].

## 3. Materials and Methods

### 3.1. Materials

All chemicals used in this study were of analytical grade. Poly(d,l-lactic-co-glycolic acid) (PLGA) (Purasorb PDLG 5004) was purchased from PURAC (Gorinchem, The Netherlands), polyvinylalcohol (PVA) with a molecular weight (MW) of 115 kDa was purchased from BDH laboratory supplies (Poole, UK), Tween 80 was purchased from MERCK (Darmstadt, Germany), phosphate buffer saline (PBS), fucoxanthin (purity >95%) were supplied by Sigma-Aldrich, dichloromethane (DCM) was purchased from Fisher Scientific (Leicestershire, UK).

### 3.2. Optimization of Medium Component by RSM

RSM was used to optimize the PS and EE of the *w*/*o*/*w* double emulsion evaporation method. A FCCCD developed by Design Expert software (version 6.0.8, Stat-Ease Inc., Minneapolis, MN, USA) [49] was used to optimize three significant extraction conditions: PVA, PLGA, and fucoxanthin concentration for optimum PS and EE. A set of 15 experimental runs with one center point (run 12) was generated. Subsequently, three different levels, low (−1), medium (0), and high (+1) were used to study the independent variables. The PS and EE were considered as the response (Y_1_) and (Y_2_), respectively. The following second-order polynomial equation explains the relationship between dependent and independent variables [49]:

Y_1_ or Y_2_ = β_0_ + β_1_*A* + β_2_*B* + β_3_*C* + β_11_*A*^2^ + β_22_*B*^2^ + β_33_*C*^2^ + β_12_*AB* + β_13_*AC* + β_23_*BC* (3)

where Y_1_ is the dependent variable (particle size, PS), and Y_2_ is the dependent variable (encapsulation efficiency, EE); *A*, *B*, and *C* are independent variables PVA, PLGA, and fucoxanthin concentration, respectively; β_0_ is an intercept term; β_1_, β_2_, and β_3_ are linear coefficients; β_12_, β_13_, and β_23_ are the interaction coefficients; and β_11_, β_22_, and β_33_ are the quadratic coefficients.

The developed regression model was evaluated by analyzing the values of regression coefficients, analysis of variance (ANOVA), *p*- and F-values (www.waset.org). The quality of fit of the polynomial model equation was expressed by the coefficient of determination, R^2^ [50,51]. Furthermore, to explain the relationship between the experimental levels of each of variables under study and the responses, the fitted polynomial equation was expressed in the form of 3D response surface and 2D contour [49].

### 3.3. Fabrication of F-LM

A double-emulsion solvent evaporation method was adopted from Mohamed and Walle [52] with some modifications. Briefly, 178 µL of dH_2_O was mixed with 22 µL of PVA, producing 0.5% *w*/*v* of aqueous phase. This aqueous phase was added into both 120 mg of PLGA (50/50) and 400 µg/mL of fucoxanthin previously dissolved in 2 mL DCM (oil phase). This mixture was homogenized at 20,500 rpm (IKA^®^ T10 basic Ultra-Turrax, Kuala Lumpur, Malaysia) for 3 min (primary emulsion, PE). After homogenization, PE was immediately subjected to 22 mL 0.5 (% *w*/*v*) PVA of 10 times the volume of PE [53]. Following this, the mixture was homogenized again at 20,500 rpm for 10 min to produce the secondary emulsion (SE). Subsequently, this SE was transferred into a continuously stirred hardening tank [53] containing 100 mL of 0.5% PVA. This stirring was continued for 2 to 3 h [54] to allow complete evaporation of DCM. The hardened microspheres were collected by centrifugation [26] (2500 rpm), washed with 600 mL distillated water and then lyophilized overnight [53]. Lyophilized microspheres were kept at −20 °C in an air-tight container with silica gel until further evaluation [53,54,55]. The general microsphere formulation recipe is shown in Table 1.

### 3.4. EE of F-LM

An accurate amount of lyophilized F-LM (2 mg) was suspended in 1 mL PBS to which 1 mL acetone was added to solubilize PLGA. The tube was centrifuged at 10,000 rpm for 3 min. The supernatant (100 µL) was transferred to CELLSTAR^®^ 96 well plate flat bottom (Greiner bio-one, Kuala Lumpur, Malaysia) [53] and analyzed using a microplate reader (Tekan/Infinite M200, NanoQuant, Kuala Lumpur, Malaysia) by measuring absorbance at 450 nm (maximum wave length for detecting fucoxanthin) [56]. The absorbance values were substituted into a standard curve of linear regression of known free fucoxanthin concentrations to obtain the actual concentrations of extracted fucoxanthin from F-LM. EE was calculated based on the ratio of the actual fucoxanthin concentration to theoretical loading, expressed as percentage [53].

### 3.5. Particle Size Analysis of F-LM

An LSP analyzer (BT-9300H, Better Size Instrument Ltd., Shanghai, China), which is a laser diffractometer was used to determine the size distribution of the microspheres prior to lyophilization, dispersing the microspheres in water until approximately 25% obscurity was reached. The size distribution was expressed as volume median diameter (VMD) [52]. Data are presented as *d* (0.5) which is equivalent volume diameter at 50% cumulative volume.

### 3.6. External Morphology of F-LM

A FE-SEM (JEOL, JSM 6700F Model) was used to capture images for evaluation of shape, size, and external morphology of the F-LM. Briefly, a small amount of lyophilized F-LM was mounted on aluminum stubs pre-pasted with double-sided copper tapes. The sample was sputter-coated with a thin layer of gold and placed inside the specimen chamber at an accelerating voltage of 3 kV at 20 °C and 10^−5^ Torr [53].

### 3.7. In Vitro Fucoxanthin Release Profile

Lyophilized F-LM (2.5 mg) was accurately weighed and added into vials containing 1 mL PBS (pH 7.4) with 0.01 % (*w*/*v*) Tween-80 to improve solubility of the drug [57]. The vials were kept at 37 °C without agitation. In vitro fucoxanthin release was assessed by intermittently sampling the vials (1 mL) at predetermined time intervals [57] (1, 3, 6, 9, and 12 h, then 1, 2, 3, and 4 days, then 1, 2, and 3 weeks, then 1 and 2 months), and was replaced with 1 mL of fresh pH 7.4 phosphate buffer [22]. The withdrawn sample was centrifuged at 10,000 rpm for 3 min. The supernatant was then collected and transferred to CELLSTAR^®^ 96 well flat bottom plate (Greiner Bio-one, Kuala Lumpur, Malaysia) [53] and read by a microplate reader (Tekan/Infinite M200, NanoQuant, Kuala Lumpur, Malaysia) with visible absorbance measurement at 450 nm (maximum wave length for detecting fucoxanthin) [56]. The amount of fucoxanthin released in each sample was determined using a curve of calibration; the reported values are averages of three replicates (n = 3). Results of in vitro fucoxanthin release studies obtained were tabulated and shown graphically as cumulative % drug release versus time [57].

### 3.8. Evaluation of Release Kinetics

The mechanism and kinetics of fucoxanthin release from F-LM was analyzed using mathematical models [58] such as zero-order kinetics, first-order kinetics and Higuchi kinetics (Table 5).

### 3.9. Degradation Study of F-LM

Degradation study method was adopted from Wang [59] with some modifications. Briefly, pre-weighed F-LMs (about 2.5 mg) were placed in individual vial tubes (15 vials) containing 1.0 mL of PBS (pH 7.4). The vial tubes were kept in an incubator that was maintained at 37 °C. At predetermined degradation intervals (0 day as control, 1 day, 1 week, 1 month, and 2 months, respectively) the F-LMs were collected by centrifugation, washed with distilled water to remove residual buffer salt, and freeze-dried overnight. Following this, the surface morphology of degraded F-LM was analyzed using FE-SEM (JEOL, JSM 6700F Model, Tokyo, Japan) at 3.0 kV [60].

## 4. Conclusions

The physical and chemical nature of fucoxanthin affects its potency and effective delivery as an anti-cancer drug against H1299 human lung cancer cells. Microencapsulation is an attractive drug delivery method to overcome this problem. In this study, fucoxanthin was successfully microencapsulated using double emulsion extraction/evaporation method to produce F-LM. The use of RSM optimized the PS and EE of the F-LM. F-LM with the best response was fabricated using a formulation of 0.5% (*w*/*v*) PVA, 6.0% (*w*/*v*) PLGA and 200 µg/mL fucoxanthin with a homogenization speed of 20,500 rpm. Under these conditions, F-LM with PS (9.18 µm) and EE (33.09%) was produced. The three factors chosen in RSM have shown significant effects on the response in PS and EE. The PS and EE in turn, affect the drug release and degradation characteristics of the F-LM. The optimization of process parameters is an important consideration in the production of drug microspheres. PS, distribution, and morphology, as well as an understanding of the kinetics and degradation pattern of the microspheres, translate into more precise control of the drug release. The information obtained from this study is important for improving efficacy and potential for commercialization of fucoxanthin as an anticancer drug in the near future.

## Figures and Tables

**Table 1:** A face centered central composite design (FCCCD) of three independent variables with their coded and actual values and one center point showing the predicted and experimental response.

Run	PVA (% *w*/*v*)	PLGA (% *w*/*v*)	Fuco (µg/mL)	PS (µm)		EE (%)	
				Ex.	Pr.*	Ex.	Pr.**
1	0.80 (+1)	6.00 (0)	200.00 (0)	5.12	4.38	28.91	28.93
2	0.50 (0)	6.00 (0)	100.00 (−1)	7.48	7.90	22.25	22.35
3	0.20 (−1)	9.00 (+1)	300.00 (+1)	6.45	6.67	33.97	33.33
4	0.20 (−1)	3.00 (−1)	100.00 (−1)	4.95	4.34	19.85	18.43
5	0.20 (−1)	9.00 (+1)	100.00 (−1)	5.18	5.22	25.57	26.85
6	0.80 (+1)	9.00 (+1)	100.00 (−1)	3.12	3.48	21.15	20.64
7	0.50 (0)	3.00 (−1)	200.00 (0)	6.85	8.04	24.56	26.40
8	0.20 (−1)	6.00 (0)	200.00 (0)	6.01	6.69	33.94	34.30
9	0.20 (−1)	3.00 (−1)	300.00 (+1)	5.36	5.02	28.72	29.13
10	0.80 (+1)	9.00 (+1)	300.00 (+1)	3.97	4.59	28.87	30.19
11	0.50 (0)	9.00 (+1)	200.00 (0)	10.95	9.71	34.87	33.41
12	0.50 (0)	6.00 (0)	200.00 (0)	9.18	9.30	33.09	32.34
13	0.80 (+1)	3.00 (−1)	300.00 (+1)	2.16	2.13	25.97	24.59
14	0.50 (0)	6.00 (0)	300.00 (+)	9.26	8.79	32.19	32.47
15	0.80 (+1)	3.00 (−1)	100.00 (−1)	2.01	1.80	10.27	10.82

PVA: Polyvinylalcohol; PLGA: Poly(d,l-lactic-co-glycolic acid); Fuco: Fucoxanthin; PS: Particle size; EE: Encapsulation efficiency; Ex.: Experiment; Pr.: Predicted. * Second order polynomial (Equaton (2)) was used to estimate the predicted response (particle size). ** Second order polynomial (Equation (3)) was used to estimate the predicted response (EE).

**Table 2:** ANOVA of quadratic model for PS (particle size).

Source	Sum of Square	F-Value	*p*-Value
Model	88.67	8.99	0.0131
PVA, *A*	13.39	12.22	0.0174
PLGA, *B*	6.96	6.35	0.0532
Fuco, *C*	1.99	1.82	0.2357
*A* ^2^	36.34	33.17	0.0022
*B* ^2^	0.46	0.42	0.5442
*C* ^2^	2.34	2.14	0.2036
*AB*	0.32	0.29	0.6121
*AC*	0.058	0.054	0.8274
*BC*	0.30	0.28	0.6208

R^2^ = 0.9418, adjusted R^2^ = 0.8371, adequate precision = 9.249, *p* < 0.05 was considered to be significant.

**Table 3:** ANOVA of quadratic model for EE (encapsulation efficiency).

Source	Sum of Square	F-value	*p*-value
Model	614.88	23.17	0.0015
PVA, *A*	72.25	24.51	0.0043
PLGA, *B*	122.92	41.69	0.0013
Fuco, *C*	256.34	86.95	0.0002
*A* ^2^	1.35	0.46	0.5287
*B* ^2^	15.24	5.71	0.0721
*C* ^2^	62.48	21.19	0.0058
*AB*	0.99	0.33	0.5879
*AC*	4.73	1.60	0.2612
*BC*	8.93	3.03	0.1424

R^2^ = 0.9766, adjusted R^2^ = 0.9344, adequate precision = 16.752, *p* < 0.05 was considered to be significant.

**Table 4:** Regression values of corresponding kinetic equation of F-LM.

Equation	Zero Order	First Order	Higuchi
R^2^	0.719	0.792	0.841

**Table 5:** Mathematical equations for the models used to describe release kinetic of drugs.

Model	Plot	Equation
Zero order	Q_t_ vs. t	Q_t_ = K_0_t
First order	ln (Q_0_ − Q_t_) vs. t	ln Q_t_ = ln Q_0_ − K_1_t
Higuchi	Q_t_ vs. t^1/2^	Q_t_ = K_h_t^1/2^
